# A Comprehensive Review of Two‐Dimensional Speckle‐Tracking Echocardiography in Assessing Right and Left Ventricular Function in Diabetic Patients

**DOI:** 10.1002/clc.70153

**Published:** 2025-05-22

**Authors:** Azin Alizadehasl, Mahshid Mokhayeri, Zeynab Sohani, Mohammad Yasin Zamanian, Parai Shahbazi, Shiva Borzouei

**Affiliations:** ^1^ Cardio‐Oncology Research Center, Rajaie Cardiovascular Medical and Research Center Iran University of Medical Sciences Tehran Iran; ^2^ Echocardiography Research Center, Rajaie Cardiovascular Medical and Research Center Iran University of Medical Sciences Tehran Iran; ^3^ Department of Endocrinology, School of Medicine Hamadan University of Medical Science Hamadan Iran; ^4^ Department of Physiology, School of Medicine Hamadan University of Medical Sciences Hamadan Iran; ^5^ Department of Cardiology, School of Medicine Hamadan University of Medical Sciences Hamadan Iran

**Keywords:** diabetes mellitus, echocardiography, HbA1c, left ventricular, right ventricular

## Abstract

**Background:**

Two‐dimensional speckle‐tracking echocardiography (2D‐STE) has emerged as a valuable tool for assessing cardiac function in patients with type 1 (T1DM) and type 2 diabetes mellitus (T2DM).

**Hypothesis:**

This review synthesizes recent studies utilizing 2D‐STE in diabetic patients, highlighting its clinical applications and findings.

**Methods:**

In this review, relevant studies were identified through comprehensive searches of major scientific databases, including PubMed, Scopus, Google Scholar, ScienceDirect, and other reputable sources.

**Results:**

The results of this study indicate that 2D‐STE is capable of detecting subclinical cardiac dysfunction in patients with both T1DM and T2DM, even in instances where conventional echocardiographic parameters appear to be within normal limits. Assessment of right ventricular (RV) function through 2D‐STE has demonstrated impaired right ventricular free wall longitudinal strain (RVFWLS) and global longitudinal strain (RVGLS) in individuals with T2DM, which correlates with suboptimal glycemic control. Furthermore, evaluation of left ventricular (LV) function has revealed decreased global longitudinal strain (GLS) and impaired LV twist mechanics in T2DM patients, particularly under conditions of physiological stress.

In T1DM patients, 2D‐STE has identified early changes in myocardial deformation, with studies reporting reduced LV and RV strain values compared to healthy controls. The technique has also been effective in assessing the impact of disease duration and glycemic control on cardiac function in both T1DM and T2DM.

**Conclusions:**

These findings underscore the potential of 2D‐STE as a sensitive and comprehensive tool for early detection of cardiac dysfunction in both T1DM and T2DM, potentially guiding management strategies and improving outcomes in these high‐risk populations.

## Graphical abstract

This figure illustrates the application of 2D speckle‐tracking echocardiography (2D‐STE) in assessing cardiac function in diabetes patients. At the center, a detailed anatomical representation of the heart is shown, highlighting the areas that can be evaluated using this advanced imaging technique. The left side of the image explains that 2D‐STE can detect subclinical cardiac dysfunction before overt clinical manifestations appear in type 2 diabetes mellitus (T2DM) patients. The figure also demonstrates that 2D‐STE shows impaired left atrial function in poorly controlled T2DM patients with coronary artery disease, as well as in those with nonalcoholic fatty liver disease (NAFLD). On the right side, the image includes a human figure representing a T2DM patient, with an illustration suggesting cellular‐level changes. The bottom right corner shows the 2D‐STE equipment used for these cardiac assessments, indicating its role in evaluating both left ventricular (LV) and right ventricular (RV) function comprehensively in diabetes patients.

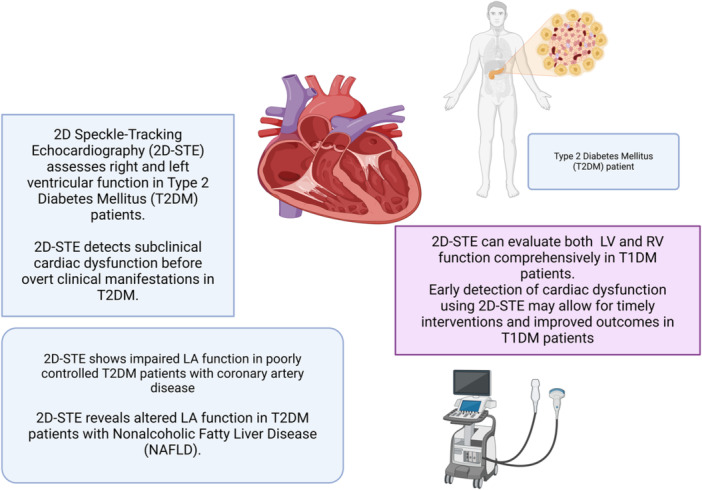

## 1 Introduction

Two‐dimensional speckle‐tracking echocardiography (2D‐STE) has emerged as a powerful tool for assessing cardiac function in various cardiovascular conditions, including diabetes mellitus (DM) [1, 2]. DM is a chronic metabolic disorder characterized by elevated blood glucose levels, which can lead to significant cardiovascular complications over time [3, 4]. The impact of DM on cardiac function is multifaceted, affecting both the left and right ventricles through various mechanisms, including metabolic disturbances, microvascular dysfunction, and autonomic neuropathy [5, 6]. Traditional echocardiographic parameters, such as ejection fraction (EF), have limitations in detecting subtle changes in cardiac function, particularly in the early stages of diabetic cardiomyopathy [7, 8]. 2D‐STE offers a more sensitive and comprehensive assessment of myocardial deformation, allowing for the detection of subclinical cardiac dysfunction before overt clinical manifestations [8]. This technique analyzes the movement of speckles within the myocardium throughout the cardiac cycle, providing quantitative measures of myocardial strain and strain rate [9]. In patients with DM, 2D‐STE has shown promise in identifying early changes in left ventricular (LV) function, even in those with preserved EF [1]. The assessment of global longitudinal strain (GLS) using 2D‐STE has been particularly valuable in detecting subtle LV dysfunction in diabetic patients [10]. Moreover, 2D‐STE has demonstrated utility in evaluating right ventricular (RV) function, which is often overlooked but can be significantly affected in DM [2]. RV dysfunction in DM can occur independently of LV dysfunction and may contribute to the overall cardiovascular risk in these patients [10]. The ability of 2D‐STE to assess both LV and RV function comprehensively makes it a valuable tool in the management of diabetic patients.

Early detection of cardiac dysfunction using 2D‐STE may allow for timely interventions and potentially improve outcomes in individuals with DM [11]. Furthermore, 2D‐STE has shown potential in monitoring the effects of various treatments, including glucose‐lowering medications and exercise interventions, on cardiac function in diabetic patients [8]. The technique's reproducibility and relatively low operator dependence make it suitable for longitudinal follow‐up of cardiac function in DM [12]. Recent studies have also explored the prognostic value of 2D‐STE‐derived parameters in predicting cardiovascular events in diabetic populations [10, 13]. The integration of 2D‐STE into routine clinical practice for diabetic patients could potentially enhance risk stratification and guide personalized management strategies. However, standardization of 2D‐STE protocols and establishment of normative values specific to diabetic populations are needed to optimize its clinical application. Additionally, the cost‐effectiveness and widespread availability of 2D‐STE in different healthcare settings remain important considerations for its broader implementation [1, 13]. Despite these challenges, the growing body of evidence supporting the use of 2D‐STE in assessing cardiac function in DM highlights its potential as a valuable clinical tool [1]. As research in this field continues to evolve, 2D‐STE may play an increasingly important role in the comprehensive cardiovascular evaluation of individuals with DM. In this review, relevant studies were identified through comprehensive searches of major scientific databases, including PubMed, Google Scholar, ScienceDirect, and other reputable sources. The search strategy involved using specific keywords related to 2D‐STE, DM, and cardiac function. Studies were selected based on their relevance to the assessment of cardiac function in patients with type 1 and type 2 diabetes mellitus using 2D‐STE. Additional inclusion criteria included original research articles in English. The titles and abstracts of retrieved articles were screened, and full texts were reviewed for eligibility. Reference lists of selected articles were also examined to identify further relevant studies. Data extraction focused on study design, population characteristics, echocardiographic techniques, and key findings related to 2D‐STE parameters in diabetic patients.

The aim of this review article is to critically evaluate the current literature on the use of 2D‐STE echocardiography in assessing right and LV function in individuals with DM, highlighting its clinical applications, advantages, and limitations.

## 2 Overview of 2D‐STE

2D‐STE emerged in the early 2000s as an extension of tissue Doppler imaging techniques. It was developed to overcome limitations of earlier methods, particularly angle dependency in assessing myocardial motion. The technique utilizes natural acoustic markers, or speckles, within the myocardium to track myocardial deformation throughout the cardiac cycle [14, 15].

This noninvasive imaging modality utilizes the natural acoustic markers, or speckles, within the myocardium to track myocardial deformation throughout the cardiac cycle. 2D‐STE provides quantitative measurements of myocardial strain and strain rate, offering a more comprehensive evaluation of cardiac mechanics compared to traditional echocardiographic parameters [16, 17].

The technique allows for the assessment of both global and regional myocardial function, making it particularly valuable in detecting subtle changes in cardiac performance [15, 18]. One of the key advantages of 2D‐STE is its ability to analyze myocardial deformation in multiple directions, including longitudinal, circumferential, and radial strains. This multi‐directional analysis provides a more complete picture of myocardial function and can reveal abnormalities that may not be apparent with conventional echocardiography [19, 20].

2D‐STE has shown particular promise in the evaluation of LV function, with GLS emerging as a robust marker of overall LV systolic function [21].

In patients with preserved EF, 2D‐STE can detect subclinical LV dysfunction, making it a valuable tool for early diagnosis of various cardiac conditions [21]. The technique has also demonstrated utility in assessing RV function, which is often challenging to evaluate using traditional echocardiographic methods [22].

RV free wall strain and RV four‐chamber longitudinal strain are two key parameters derived from 2D‐STE that provide insights into RV performance [23].

2D‐STE has found applications across a wide range of cardiovascular conditions, including ischemic heart disease, cardiomyopathies, and valvular heart disease. In the field of congenital heart disease, 2D‐STE has proven valuable in assessing ventricular function in pediatric patients, offering a more sensitive approach than conventional methods [24].

The technique has also been applied to evaluate cardiac function in various systemic diseases that can affect the heart, such as beta‐thalassemia major [16].

One of the strengths of 2D‐STE is its relatively low operator dependence and good reproducibility, making it suitable for longitudinal follow‐up of patients. The technique has shown promise in monitoring the effects of various interventions, including medical therapies and cardiac rehabilitation programs [25].

In sports cardiology, 2D‐STE has been used to assess the impact of different levels of exercise on myocardial performance, helping to distinguish between physiological adaptations and pathological changes [25].

Despite its many advantages, 2D‐STE does have some limitations, including dependence on image quality and the need for standardization across different ultrasound systems and software platforms [14, 26].

Ongoing research is focused on addressing these challenges and further refining the technique. As 2D‐STE continues to evolve, it is increasingly being integrated into routine clinical practice, complementing traditional echocardiographic assessments and providing additional diagnostic and prognostic information. The growing body of evidence supporting the use of 2D‐STE in various cardiac conditions underscores its potential to become a standard tool in comprehensive cardiovascular evaluation.

## 3 Recent Developments

In recent years, 2D‐STE has been increasingly used for:

Endocrine disorders: Detecting subclinical LV dysfunction in patients with acromegaly [27, 28].

Cardiotoxicity monitoring: Detecting early signs of cardiac dysfunction in cancer patients undergoing chemotherapy [29, 30].

Thyroid disorders: Assessing cardiac contractility in patients with subclinical hyperthyroidism [31].

## 4 Using the 2D‐STE to Assess Right and Left Ventricular Function in Patients With Type 2 Diabetes Mellitus (T2DM)

2D‐STE has emerged as a powerful tool for assessing myocardial function in patients with T2DM. This advanced echocardiographic technique allows for the quantitative evaluation of both left and right ventricular function by tracking the motion of speckles in the myocardium throughout the cardiac cycle. 2D‐STE offers several advantages over conventional echocardiographic parameters in evaluating cardiac function in T2DM patients. It provides a more sensitive and accurate assessment of subtle myocardial changes, allowing for the detection of subclinical dysfunction before overt clinical manifestations. This is particularly important in T2DM patients, where early identification of cardiac abnormalities can guide timely interventions and potentially improve outcomes.

For LV assessment, 2D‐STE enables the measurement of global and regional longitudinal, circumferential, and radial strain, offering a comprehensive evaluation of LV mechanics. In T2DM patients, LV longitudinal strain has been shown to be impaired even in the presence of normal EF, indicating its value in detecting early diabetic cardiomyopathy.

RV function assessment, traditionally challenging due to its complex geometry, has been significantly improved with 2D‐STE. The technique allows for the evaluation of RV free wall and septal strain, providing insights into RV mechanics that were previously difficult to obtain.

Recent studies have demonstrated that RV dysfunction, as assessed by 2D‐STE, is present in T2DM patients and may have prognostic implications.

By utilizing 2D‐STE for both LV and RV assessment in T2DM patients, clinicians can gain a more comprehensive understanding of cardiac function, potentially leading to earlier detection of diabetic cardiomyopathy and improved management strategies.

This introduction sets the stage for a detailed exploration of the application and significance of 2D‐STE in evaluating biventricular function in T2DM patients.

HbA1c or glycated hemoglobin, is a critical marker used to assess long‐term blood glucose control in individuals, particularly those with diabetes [32].

The relationship between HbA1c levels and RV free wall longitudinal strain (RVFWLS) is significant in the context of T2DM [2]. Higher HbA1c levels are independently associated with subclinical RV dysfunction, indicating that poor glycemic control adversely affects RV performance. Specifically, RVFWLS is a sensitive measure that reflects the impact of hyperglycemia on the RV myocardium [2].

Zhang et al. investigated the association between glycemic control and RV function in individuals with T2DM using 2D‐STE and three‐dimensional echocardiography (3DE). The study results revealed that patients with uncontrolled T2DM exhibited reduced RVFWLS compared to those with controlled blood glucose levels. This reduction in RVFWLS was more pronounced in the uncontrolled subgroup, indicating a significant impact of poor glycemic control on RV function. Additionally, the uncontrolled T2DM subgroup showed higher pulmonary artery systolic pressure (PASP) and a lower RVFWLS/PASP ratio, suggesting increased RV afterload. The incidence of RV dysfunction was significantly higher in the uncontrolled T2DM patients compared to the controlled subgroup. Furthermore, the study demonstrated an impaired right ventricular‐pulmonary circulation (RV‐PC) coupling in diabetes, highlighting the importance of effective glycemic control for preventing diabetic cardiomyopathy. These findings underscore the critical role of maintaining controlled blood glucose levels to mitigate cardiovascular complications in T2DM patients [2].

RVGLS has emerged as a key parameter for assessing the systolic function of the RV, offering several advantages over conventional echocardiographic measures [33]. It is angle‐independent, less load‐dependent than other parameters, and highly accurate for measuring regional myocardial deformation, allowing for the detection of subclinical RV dysfunction even when conventional parameters appear normal [33]. RVGLS has demonstrated strong prognostic value across various cardiovascular conditions, including pulmonary hypertension, heart failure (HF), and cardiac amyloidosis [34, 35, 36]. Studies have shown that RVGLS correlates better with cardiac MRI‐derived RV EF compared to parameters like tricuspid annular plane systolic excursion (TAPSE) or fractional area change (FAC), and provides incremental prognostic information beyond LV EF in HF patients [34, 37].

Wu et al. utilized 2D‐STE to assess RV systolic function in individuals with prediabetes and T2DM. Results indicated that the RVGLS and RV free wall strain (RVFW‐LS) decreased progressively from normoglycemic controls to prediabetic and diabetic patients. This decline in strain values suggests early impairment of RV systolic function in patients with abnormal glucose metabolism. The study found that 2D‐STE was more sensitive than conventional echocardiography in detecting these subtle changes. Multivariate regression analysis identified HbA1c, interventricular septal longitudinal strain (IVS‐LS), and LV end‐diastolic diameter (LVEDd) as independent predictors of RVGLS. The findings highlight the potential of 2D‐STE in identifying early cardiac dysfunction, which might not be evident through conventional methods. Overall, the study underscores the importance of monitoring glucose levels to mitigate myocardial damage [10].

Epicardial adipose tissue (EAT) is a unique fat depot located between the myocardium and visceral pericardium, sharing the same microcirculation as the adjacent myocardium [38, 39]. Under normal physiological conditions, EAT serves protective functions, including acting as a buffer against mechanical stress, providing thermal regulation, and serving as a local energy source for the heart [38]. However, in pathological states, particularly in patients with T2DM, increased EAT thickness has been associated with adverse cardiovascular outcomes [40]. Studies have shown that patients with T2DM tend to have higher EAT volume compared to nondiabetic individuals, and this increased EAT is linked to a higher risk of coronary artery disease (CAD), metabolic syndrome, and overall cardiovascular morbidity and mortality [41, 42].

Systolic blood pressure (SBP) and HbA1c levels are crucial clinical parameters in the management of patients with T2DM. Studies have identified specific threshold effects between albumin/creatinine ratio (ACR) and both HbA1c and SBP, with risk threshold values of HbA1c = 6.4% and SBP = 127 mmHg, respectively, above which the logarithm function (LnACR) increased dramatically [43]. Higher levels of HbA1c have been associated with an increased risk of hypertension (HT), even after adjusting for various confounding factors, suggesting a potential causal relationship between glycemic control and blood pressure regulation [44]. Furthermore, long‐term visit‐to‐visit variability in both HbA1c and SBP has been shown to represent a combined and additive risk for cardiovascular disease (CVD) incidence in patients with T2DM, with a possible synergistic effect between HbA1c variability and mean SBP levels [45].

Song et al. utilized 2D‐STE to assess RV function in patients with T2DM. The study found that EAT thickness was significantly higher in patients with T2DM compared to nondiabetic controls. In T2DM patients, those with EAT thickness of 5 mm or more exhibited more pronounced RV dysfunction. Specifically, the right ventricular longitudinal strain (RV‐LS) and early‐diastole longitudinal strain rate (RV LSR‐E) were significantly lower in T2DM patients with thicker EAT. The study demonstrated that EAT was negatively correlated with RV LS and RV LSR‐E, independent of other cardiovascular risk factors such as SBP and HbA1c levels. These findings suggest that increased EAT is associated with subclinical RV systolic and early diastolic dysfunction in T2DM patients. The use of 2D‐STE was effective in detecting these subtle myocardial dysfunctions. Overall, the study highlights the potential role of EAT as a cardiovascular risk factor in T2DM, warranting further investigation and potential therapeutic targeting.

Song et al. utilized 2D‐STE to assess the relationship between EAT and LV function in patients with T2DM. The results indicated that patients with a GLS of less than 18% exhibited higher age, body mass index (BMI), waist circumference (WC), SBP, diastolic blood pressure (DBP), low‐density lipoprotein cholesterol (LDL‐C), HbA1c, E/e ratio, and EAT thickness compared to those with GLS of 18% or more. The study found no significant differences in other echocardiographic parameters such as left atrial diameter (LAD), interventricular septal thickness (IVSD), LV posterior wall thickness (LVPWD), LVEDD, LV end‐systolic diameter (LVESD), and LV ejection fraction (LVEF) between the two groups. Multivariate linear regression analysis revealed that EAT thickness was independently associated with both systolic and diastolic dysfunction of the left ventricle in T2DM patients. The study demonstrated good reliability in the measurement of GLS, EAT, and E/e, with an intra‐class correlation coefficient (ICC) of 0.75 or higher. The findings suggest that thickened EAT is linked to impaired LV function in T2DM patients. This association underscores the potential of EAT as a therapeutic target for improving cardiac function in this population.

Nonalcoholic fatty liver disease (NAFLD) is increasingly recognized as the most common chronic liver disease among patients with T2DM [46]. The diagnosis rate of NAFLD is rising, and T2DM patients are at a higher risk of developing severe forms of this condition [47]. Furthermore, NAFLD can lead to CVDs, highlighting the importance of monitoring cardiac function in these patients.

Chang et al. utilized 2D‐STE to evaluate LA function in patients with T2DM and NAFLD. It found that patients with moderate to severe NAFLD exhibited significant changes in LA function compared to those with mild or no fatty liver disease. Specifically, the LA active ejection fraction (LAAEF) and late diastolic strain rates (LASRa) were significantly higher in patients with severe NAFLD. Conversely, the LA passive ejection fraction (LAPEF), SRes, and systolic strain rates (LASRs) were notably decreased in this group. These findings suggest that as NAFLD severity increases, there are marked alterations in LA function, potentially indicating early cardiac dysfunction. The study underscores the importance of monitoring cardiac function in T2DM patients with NAFLD, as early changes in LA function could serve as an indicator for intervention and management. Overall, 2D‐STE proved to be a valuable noninvasive tool for detecting early changes in LA function in this patient population [12].

Global peak systolic longitudinal rotation (PSLR) is a sensitive measure of LV systolic function that can detect subclinical LV dysfunction even when conventional parameters like EF appear normal [37, 48]. Importantly, HbA1c levels have been found to negatively correlate with apex and global PSLR in T2DM patients, indicating that those with higher HbA1c values demonstrate larger clockwise apex and global PSLR [48]. This relationship suggests that PSLR could serve as a sensitive indicator of LV systolic dysfunction in T2DM patients, potentially reflecting the impact of poor glycemic control on cardiac function [48, 49].

Huang et al. investigated the use of 2D‐STE to assess LV systolic dysfunction in patients with T2DM. It found that T2DM patients exhibited a significant clockwise PSLR, whereas normal subjects showed minimal PSLR. A negative correlation was observed between HbA1c levels and both apex and global PSLR, indicating that higher HbA1c levels were associated with greater PSLR and impaired cardiac function. The study demonstrated that PSLR could effectively detect LV systolic dysfunction in T2DM patients. The reproducibility and repeatability of PSLR measurements were confirmed, with no significant differences found in interobserver and intraobserver variability. The study concluded that PSLR is a reliable and convenient tool for detecting cardiac dysfunction in T2DM patients. Additionally, HbA1c was identified as a potential predictor of systolic dysfunction in this population [48].

Dobutamine stress echocardiography (DSE) is a diagnostic imaging technique used to evaluate cardiac function, particularly in patients who are unable to undergo traditional exercise stress testing [50]. During DSE, dobutamine, a medication that stimulates the heart, is administered to increase heart rate and mimic the effects of exercise. This allows for the assessment of myocardial function and the detection of wall motion abnormalities that may not be visible at rest [51].

DSE is particularly useful in diagnosing CAD and can help reveal subclinical abnormalities in myocardial mechanics, especially in asymptomatic patients with conditions like T2DM. The technique enables clinicians to observe changes in LV function and to assess the heart's response to increased workload, providing valuable information for early diagnosis and management of cardiac conditions [52, 53].

Philouze et al. utilized 2D‐STE to assess myocardial mechanics in patients with T2DM and control subjects. At rest, there were no significant differences in systolic longitudinal strain (LS) and LS rate between the two groups. However, under dobutamine stress, significant differences emerged, indicating impaired myocardial functional reserve in T2DM patients. Specifically, T2DM patients exhibited reduced apical rotation and twist mechanics compared to controls during stress. These alterations were not apparent at rest, highlighting the utility of stress echocardiography in unmasking subtle myocardial dysfunction. The study also found that EAT was a significant contributor to these stress‐induced changes. Furthermore, the speckle‐tracking echocardiographic indexes significantly increased in both groups in response to dobutamine infusion, except for apical rotation, which did not change in the diabetic population. Overall, the results underscore the importance of using stress echocardiography to detect early myocardial dysfunction in asymptomatic T2DM patients [53].

Fragmented QRS (fQRS) is an electrocardiographic finding characterized by the presence of additional deflections or notches in the QRS complex of the ECG [54]. It is considered a marker of myocardial fibrosis or scar tissue and is associated with an increased risk of cardiovascular adverse events [55]. fQRS can indicate underlying cardiac issues, such as myocardial scarring, and has been linked to various conditions, including diabetic cardiomyopathy, CAD, and HF [56, 57].

Bayramoğlu et al. utilized 2D‐STE to assess LV function in patients with T2DM and fQRS complexes. The results demonstrated statistically significant differences in LV strain values between patients with and without fQRS, indicating subclinical LV dysfunction in those with fQRS. Specifically, the LV‐GLS was significantly lower in the fQRS‐positive group compared to the fQRS‐negative group. The study identified fQRS and the duration of diabetes as independent predictors of LV‐GLS, suggesting that fQRS is a reliable marker for detecting early cardiac involvement in diabetic patients. Additionally, the study found that fQRS and minimum left atrial volume index (minLAVI) were independent predictors of the E/SRivr ratio, an index of LV filling pressure. The findings suggest that fQRS could serve as an indicator of diabetic cardiomyopathy, highlighting the potential of STE in evaluating myocardial function in diabetic patients. Overall, the study emphasizes the importance of identifying fQRS in routine clinical assessments to predict better and manage cardiac complications in diabetes [58].

Early diastolic strain (EDS) and early diastolic strain rate (SRe) are important indices used to assess LA function, providing valuable insights into the heart's diastolic performance [59]. These parameters, measured through advanced echocardiographic techniques such as speckle tracking, offer a more sensitive and accurate evaluation of LA mechanics compared to traditional volumetric measurements [59]. EDS and SRe reflect the LA's ability to expand and receive blood from the pulmonary veins during early ventricular diastole, serving as early indicators of diastolic dysfunction even before overt clinical symptoms manifest [60, 61]. Recent studies have demonstrated that impaired EDS and SRe are associated with various cardiovascular conditions, including HT, obesity, and HF, highlighting their potential as prognostic markers in clinical practice [62, 63].

Hosseinsabet et al. utilized 2D‐STE to assess LA function in CAD patients with varying levels of T2DM control. The results indicated that EDS and SRe, markers of LA conduit function, were significantly lower in CAD patients with poorly controlled diabetes compared to those with well‐controlled diabetes and euglycemic controls. This impairment in LA function was identified as an independent determinant in patients with poorly controlled diabetes. The study found no significant differences in LA volumetric parameters among the groups, suggesting that the observed functional impairments were not due to changes in atrial size. Multivariable analysis confirmed that poorly controlled diabetes, along with factors like sex and hematocrit, were independent determinants of the impaired indices. The study highlights the importance of glycemic control in preserving cardiac function in diabetic patients with CAD. Overall, the findings underscore the detrimental impact of poor diabetes management on cardiac health, particularly in the context of CAD [64].

Global peak atrial longitudinal strain (PALS) has emerged as a valuable echocardiographic parameter for assessing LA dysfunction in patients with T2DM and HF. This advanced imaging technique, utilizing speckle tracking echocardiography, provides a more sensitive and accurate evaluation of LA mechanics compared to conventional measures, allowing for earlier detection of subtle changes in atrial function [65]. In patients with T2DM and HF, reduced global PALS values have been associated with worse cardiovascular outcomes and increased risk of atrial fibrillation [65, 66].

Peak atrial contraction strain (PACS) has emerged as a valuable echocardiographic parameter for assessing LA dysfunction in patients with T2DM. This advanced imaging technique, utilizing speckle tracking echocardiography, provides a more sensitive and accurate evaluation of LA contractile function compared to conventional measures, allowing for earlier detection of subtle changes in atrial mechanics [65]. In patients with T2DM, reduced PACS values have been associated with impaired LA booster pump function and increased risk of cardiovascular complications, including atrial fibrillation and HF [65, 67]. Recent studies have demonstrated that PACS, along with other LA strain parameters such as PALS, can serve as independent predictors of adverse cardiovascular events in T2DM patients, highlighting their potential as prognostic tools in clinical practice and guiding treatment decisions [65, 68].

Georgievska‐Ismail et al. investigated the role of 2D‐STE in assessing LA dysfunction in patients with T2DM and HF with preserved ejection fraction (HFpEF). It found that global PALS and PACS were significantly lower in diabetic patients compared to nondiabetic patients. These reduced strain values were associated with a higher prevalence, greater severity, and longer duration of diabetes. Multiple linear regression analysis identified diabetes as an independent predictor of reduced global PALS and PACS. The study demonstrated that LA strain measurements using 2D‐STE provided additional value over traditional echocardiographic parameters in detecting LA dysfunction. The inclusion of LA strain parameters significantly improved the discrimination between patients with and without diabetes. Overall, the findings suggest that 2D‐STE is a valuable tool for early detection of LA dysfunction in diabetic patients with HFpEF [69].

Left atrial strain parameters provide valuable insights into the mechanical properties and performance of the left atrium during different phases of the cardiac cycle. LA_S‐S_, or systolic left atrial strain, represents the peak LS of the left atrium during ventricular systole, corresponding to the reservoir function of the LA and measuring its ability to expand and store blood during ventricular contraction. LA_SR‐S_, or systolic left atrial strain rate, quantifies the rate of deformation of the LA during ventricular systole, providing information about the speed at which the LA expands to accommodate blood flow from the pulmonary veins. L_AS‐E_, or early diastolic left atrial strain, reflects the LA strain during early ventricular diastole, corresponding to the conduit function of the LA and measuring the passive emptying of the LA as the left ventricle relaxes. Lastly, LA_SR‐E_, or early diastolic left atrial strain rate, quantifies the rate of LA deformation during early ventricular diastole, indicating how quickly the LA empties passively into the left ventricle [70, 71, 72].

Liu et al. utilized 2D‐STE to assess LA phasic function in patients with HT and T2DM. The results indicated that both the HT group and the HT with diabetes (HT + DM) group exhibited significantly lower peak LA_S‐S_ and LA_SR‐S_ compared to the control group. Specifically, the LA_S‐E_ and LA_SR‐E_, which reflect early diastolic function, were notably reduced in the HT group and further decreased in the HT + DM group. This suggests that HT impairs LA reservoir and conduit functions, while the presence of diabetes exacerbates the conduit dysfunction. Multivariate regression analysis confirmed that both HT and diabetes independently contributed to the observed reductions in LA_S‐E_ and LA_SR‐E_. These findings underscore the sensitivity of 2DSTE‐derived strain and strain rate imaging in detecting subtle changes in LA function associated with these conditions [73].

Table 1 summarizes various studies that used 2D‐STE to assess cardiac function in patients with T2DM and related conditions. It provides details on the total number of participants, the number of T2DM patients, control or other groups, subgroups within the studies, and key results, highlighting the effectiveness of 2D‐STE in detecting subtle cardiac dysfunctions and the impact of factors such as glycemic control, EAT thickness, and NAFLD on cardiac function in T2DM patients.

**Table 1:** Summary of studies using 2D‐STE to assess cardiac function in patients with T2DM.

Study	Total participants	T2DM patients	Control/other groups	Subgroups	Key results
Zhang et al.	291	207	84 NGM	91 controlled T2DM (HbA1c < 7.0%), 116 uncontrolled T2DM (HbA1c ≥ 7.0%)	Uncontrolled T2DM patients showed reduced RVFWLS, higher PASP, and lower RVFWLS/PASP ratio compared to controlled T2DM patients.
Wu et al.	144	52	49 NG, 43 PDM	N/A	RVGLS and RVFW‐LS decreased progressively from the NG to the PDM to the T2DM groups. HbA1c, IVS‐LS, and LVEDd were independent predictors of RVGLS.
Song et al. (EAT study)	224	154	70 non‐T2DM	77 T2DM with EAT < 5 mm, 69 T2DM with EAT ≥ 5 mm	T2DM patients with EAT ≥ 5 mm showed more pronounced RV dysfunction, with lower RV‐LS and RV LSR‐E.
Song et al. (GLS study)	116	116	N/A	53 with GLS < 18%, 63 with GLS ≥ 18%	Patients with GLS < 18% had higher age, BMI, WC, SBP, DBP, LDL‐C, HbA1c, E/e ratio, and EAT thickness. EAT thickness was independently associated with LV dysfunction.
Chang et al.	97	97	N/A	Groups A, B, and C based on NAFLD severity	Patients with moderate to severe NAFLD showed higher LAAEF and LASRa, and lower LAPEF, LASRe, and LASRs.
Huang et al.	103	51	52 normal controls	N/A	T2DM patients showed significant clockwise PSLR. HbA1c levels negatively correlated with apex and global PSLR.
Philouze et al.	79	44	35 healthy controls	N/A	T2DM patients showed impaired myocardial functional reserve under dobutamine stress, with reduced apical rotation and twist mechanics.
Bayramoğlu et al.	178	178	N/A	50 fQRS positive, 128 fQRS negative	fQRS‐positive group showed lower LV‐GLS. fQRS and diabetes duration were independent predictors of LV‐GLS.
Hosseinsabet et al.	110	66	44 euglycemic	33 WCBS, 33 PCBS	PCBS patients showed lower EDS and SRe compared to WCBS and euglycemic groups.
Georgievska‐Ismail	218	108	110 non‐DM HFpEF	N/A	49.5% of HFpEF patients had T2DM. The cohort primarily consisted of individuals over 60 years with a high prevalence of cardiovascular risk factors.
Liu et al.	190	65	99 HT, 26 healthy controls	N/A	The study compared HT patients with and without T2DM, but specific results were not provided in the given information.

Abbreviations: EAT = epicardial adipose tissue, fQRS = fragmented QRS, GLS = global longitudinal strain, HFpEF = heart failure with preserved ejection fraction, HT = hypertension, NAFLD = nonalcoholic fatty liver disease, NG = normoglycemic, NGM = normal glucose metabolism, PCBS = poorly controlled blood sugar, PDM = prediabetics, WCBS = well‐controlled blood sugar.

## 5 Using the 2D‐STE to Assess Right and Left Ventricular Function in Patients With Type 1 Diabetes Mellitus (T1DM)

2D‐STE has emerged as a powerful tool for evaluating cardiac function in patients with T1DM [74]. This advanced imaging technique allows for the quantitative assessment of myocardial deformation and strain, providing valuable insights into both left and RV mechanics [9, 74]. In patients with T1DM, 2D‐STE can detect subclinical changes in cardiac function before conventional echocardiographic parameters show abnormalities [9]. The ability to identify early cardiac dysfunction is crucial, as it may allow for timely interventions to prevent or delay the progression of diabetic cardiomyopathy [75]. Studies have shown that 2D‐STE can reveal impaired LV‐GLS in T1DM patients, even when EF remains normal. This reduction in LV‐GLS is often one of the earliest signs of diabetic cardiomyopathy and may precede changes in other strain parameters [9, 75]. RV function can also be assessed using 2D‐STE, providing a comprehensive evaluation of cardiac performance in T1DM patients [74]. Research has demonstrated that RVFWLS may be reduced in T1DM patients compared to healthy controls, indicating subclinical RV dysfunction. The assessment of both LV and RV function using 2D‐STE allows for a more complete understanding of the cardiac effects of T1DM [9].

These four studies all used 2D‐STE to assess cardiac function, particularly right and/or LV function, in patients with T1DM. Their common goal was to detect subclinical cardiac dysfunction in T1DM patients using this advanced echocardiographic technique.

Ahmed et al. utilized 2D‐STE to assess RV function in young Egyptians with T1DM. It revealed that these patients exhibited significant early RV systolic dysfunction, despite having normal RV and LV ejection fractions. The 2D‐STE was effective in detecting subclinical cardiac changes, specifically a notable decrease in RV‐GLS among the diabetic group compared to healthy controls. Additionally, the study found prevalent RV diastolic dysfunction in T1DM patients, with significant differences in echocardiographic parameters such as the E/A and E/Em ratios. The research identified higher BMI and RV E velocity as independent predictors of RV dysfunction, suggesting potential areas for intervention. These findings underscore the importance of early echocardiographic screening in T1DM patients to detect subclinical RV dysfunction. The study highlights the need for timely lifestyle and medical interventions to prevent or delay HF development in this population [74].

Berceanu et al. utilized 2D‐STE to evaluate LV and RV function in young adults with DM1. The results indicated that while there were no significant differences in LVEF between the DM1 group and healthy controls, the DM1 group exhibited reduced LS in the LV endocardium and myocardium. Specifically, the GLS for the endocardium and myocardium was significantly lower in the DM1 group compared to controls. Additionally, the study found that mechanical dispersion was higher in the diabetes group, suggesting increased electrical heterogeneity. Despite these differences, RV strain measurements did not show significant differences between the groups. The findings highlight subclinical myocardial dysfunction in DM1 patients, characterized by lower LV endocardial and myocardial strain and higher mechanical dispersion. This suggests that even in the absence of overt heart disease, young adults with DM1 may have underlying cardiac dysfunction detectable through advanced imaging techniques like STE [9].

Rakha et al. utilized 2D‐STE to assess LV function in pediatric patients with long‐standing T1DM. It was found that the mean LV‐GLS was significantly lower in diabetic patients compared to the control group, with values of −19.7% versus −21.8%, respectively, and a *p*‐value of 0.038. Additionally, the mean LV global circumferential strain (GCS) was also significantly reduced in the diabetic group, measuring −21.3% compared to −27.1% in controls, with a *p*‐value of 0.001. Some specific segments, such as the mid‐anterior, anterolateral, apical, and anterior segments, were more significantly affected in the diabetic group. Despite these findings, the EF derived from 2D speckle tracking did not show a statistically significant difference between the diabetic and control groups. The study highlighted that the mean HbA1c level was a significant predictor of decreased GLS in diabetic children. Overall, the results underscore the utility of 2D speckle tracking in detecting subclinical myocardial changes in pediatric patients with T1DM, even when conventional echocardiographic measures appear normal [75].

Łukawska‐Tatarczuk et al. utilized 2D‐STE to assess LV function in women with T1DM, focusing on those with positive antithyroid peroxidase antibodies (aTPO+). The results revealed that the T1DM aTPO+ group exhibited significantly lower GLS values compared to both the T1DM aTPO– group and healthy controls, indicating impaired myocardial function. Specifically, the lowest LS values were observed in the inferoseptal and anterolateral segments in the four‐chamber (4CH) view, with significant differences between the T1DM aTPO+ group and controls. Additionally, the two‐chamber (2CH) view showed significant differences in strain values between the T1DM aTPO+ and T1DM aTPO– groups. The study also found that both T1DM groups had significantly lower anterolateral wall strain compared to controls, while only the T1DM aTPO+ group showed significantly lower anterior wall strain. These findings suggest that thyroid autoimmunity may contribute to early myocardial dysfunction in T1DM, particularly affecting specific LV segments. The study highlights the potential of STE as a sensitive tool for detecting subclinical cardiac dysfunction in this population [13].

Table 2 summarizes various studies that used 2D‐STE to assess cardiac function in patients with T1DM, providing details on the total number of participants, the number of T1DM patients, control groups, and key results for each study.

**Table 2:** Summary of studies using 2D‐STE to assess cardiac function in patients with T1DM.

Study	Total participants	T1DM patients	Control group	Key results
Ahmed et al.	54	39 (13 M, 26 F)	15 (5 M, 10 F)	T1DM patients showed significantly lower LV and RV GLS compared to controls. LV GLS correlated negatively with HbA1c levels and diabetes duration.
Berceanu et al.	130	50 (26 M, 24 F)	80 (55 M, 25 F)	T1DM patients exhibited lower LV GLS and higher E/e' ratio compared to controls. RV free wall strain was also reduced in T1DM patients.
Rakha et al.	83	48	35	T1DM patients had significantly lower LV and RV GLS compared to controls. LV GLS correlated negatively with HbA1c levels and diabetes duration.
Łukawska‐Tatarczuk et al.	88	59 (all F)	29 (all F)	T1DM patients with positive aTPO showed lower LV GLS compared to T1DM patients with negative aTPO and controls. RV function was also impaired in T1DM patients.

Abbreviations: aTPO = antithyroid peroxidase antibody, F = female, GLS = global longitudinal strain, HbA1c = glycated hemoglobin, LV = left ventricular, M = male, RV = right ventricular.

## 6 Using the 2D‐STE to Assess Right and Left Ventricular Function in Patients With Gestational Diabetes Mellitus (GDM)

GDM is defined as glucose intolerance first recognized during pregnancy. Recent global estimates indicate a prevalence of approximately 14%–16%, and this rate is expected to continue rising in the coming years [76, 77]. 2D‐STE is a sensitive imaging technique used to detect subclinical cardiac dysfunction in patients with GDM [78, 79]. Li et al. demonstrated that LV function in women with GDM was assessed using both conventional echocardiography and 2D‐STE. The results indicated that while conventional echocardiographic parameters and EF were similar between GDM patients and healthy controls, the LV‐GLS was significantly reduced in the GDM group. Notably, 66% of GDM patients had an absolute LV‐GLS of less than 20%, suggesting subclinical systolic dysfunction despite preserved EF. Furthermore, GDM patients showed significantly lower LA reservoir and conduit strain values, indicating early diastolic function impairment, although LA contractile function did not differ between the groups. Multivariate regression analysis confirmed that both LV‐GLS and LA conduit strain were independently associated with GDM. These findings suggest that 2D‐STE is more sensitive than conventional echocardiography in detecting early, subclinical myocardial dysfunction in pregnant women with GDM [78]. In another study, Sonaglioni et al. demonstrated that women with GDM experience significant subclinical impairment in both ventricular and atrial myocardial function, as assessed by 2D‐STE, compared to women with uncomplicated pregnancies. Specifically, women with GDM exhibited higher rates of overweight and obesity, increased LV mass, elevated filling pressures, and higher blood pressure, while maintaining a preserved EF. This indicates that conventional measures may overlook early dysfunction. The study revealed that myocardial strain indices—including left and right ventricular global LS and both atrial reservoir strains—were significantly lower in women with GDM, with these impairments persisting in about one‐third of cases postpartum. Moreover, it found that a higher BMI during pregnancy was the strongest independent predictor of persistent LV strain impairment after delivery, with a BMI of ≥ 30 kg/m² showing nearly perfect sensitivity and specificity for identifying at‐risk women. These findings suggest that even mild hyperglycemia in GDM, particularly in conjunction with obesity, can lead to early and persistent subclinical cardiac dysfunction [79]. The results underscore the importance of advanced echocardiographic techniques for early detection and the potential need for targeted postpartum cardiovascular risk management in this population.

## 7 Future Directions

The comprehensive review of 2D‐STE in assessing cardiac function in diabetic patients has revealed several promising avenues for future research.

### 7.1 Longitudinal Studies

Long‐term follow‐up studies are needed to evaluate the prognostic value of 2D‐STE parameters in predicting cardiovascular outcomes in both T1DM and T2DM patients.

### 7.2 Standardization

Efforts should be made to establish standardized protocols and reference values for 2D‐STE measurements specific to diabetic populations, accounting for age, gender, and diabetes duration.

### 7.3 Integration With Other Modalities

Future research should explore the combination of 2D‐STE with other imaging modalities, such as cardiac MRI or CT, to provide a more comprehensive assessment of cardiac structure and function in diabetic patients.

### 7.4 Therapeutic Interventions

Studies investigating the impact of various therapeutic interventions (e.g., novel antidiabetic medications, lifestyle modifications) on 2D‐STE parameters could help guide personalized treatment strategies.

### 7.5 Machine Learning Applications

Developing machine learning algorithms to analyze 2D‐STE data could enhance the technique's diagnostic and prognostic capabilities in diabetic cardiomyopathy.

### 7.6 Three‐Dimensional STE

Further exploration of three‐dimensional STE could provide additional insights into cardiac mechanics in diabetic patients, potentially offering even greater sensitivity in detecting subclinical dysfunction.

## 8 Limitations

Despite the promising results, several limitations should be considered.

### 8.1 Image Quality Dependence

The accuracy of 2D‐STE measurements is highly dependent on image quality, which can be challenging in certain patient populations (e.g., obese individuals).

### 8.2 Lack of Standardization

The absence of universally accepted reference values and standardized protocols across different ultrasound systems and software platforms limits the comparability of results between studies.

### 8.3 Technical Expertise

2D‐STE requires specialized training and expertise, which may limit its widespread adoption in clinical practice.

### 8.4 Cost and Availability

The technology and software required for 2D‐STE may not be readily available in all healthcare settings, particularly in resource‐limited areas.

### 8.5 Limited Long‐Term Data

There is a scarcity of long‐term follow‐up studies evaluating the prognostic value of 2D‐STE parameters in diabetic populations1.

### 8.6 Confounding Factors

The impact of comorbidities common in diabetic patients (e.g., HT, obesity) on 2D‐STE parameters needs further clarification.

### 8.7 Reproducibility Concerns

While 2D‐STE has shown good reproducibility in many studies, inter‐vendor variability and the potential for measurement errors in less experienced hands remain concerns.

Addressing these limitations and pursuing the outlined future directions will help solidify the role of 2D‐STE in the comprehensive cardiovascular evaluation and management of patients with DM.

## 9 Conclusion

2D‐STE has emerged as a powerful and sensitive tool for assessing cardiac function in patients with both T2DM and T1DM. This review highlights the significant contributions of 2D‐STE in detecting subclinical cardiac dysfunction, evaluating both left and right ventricular performance, and assessing the impact of various factors on myocardial function in diabetic patients. The ability of 2D‐STE to detect subtle changes in myocardial mechanics, even when conventional echocardiographic parameters appear normal, underscores its value in early diagnosis and risk stratification for both T2DM and T1DM patients. Key findings from the reviewed studies demonstrate the utility of 2D‐STE in assessing the relationship between glycemic control and cardiac function, evaluating the impact of EAT on myocardial performance, and detecting early changes in left atrial function across both types of diabetes. The technique has also shown promise in unmasking subclinical myocardial dysfunction during stress conditions and in identifying cardiac abnormalities associated with fQRS complexes in diabetic patients. Furthermore, 2D‐STE has demonstrated potential in evaluating left atrial dysfunction in diabetic patients with HF with preserved EF, providing additional prognostic information for both T1DM and T2DM populations. As research in this field continues to evolve, 2D‐STE is likely to play an increasingly important role in the comprehensive cardiovascular evaluation and management of individuals with both types of DM, potentially guiding personalized treatment strategies and improving long‐term outcomes.

## Ethics Statement

The authors have nothing to report.

## Consent

The authors have nothing to report.

## Conflicts of Interest

The authors declare no conflicts of interest.

## Data Availability

The data used to support the findings of this study are available from the corresponding author upon request.

## References

[1] X.‐T. Song , S. K. Wang , P. Y. Zhang , L. Fan , and Y. F. Rui , “Association Between Epicardial Adipose Tissue and Left Ventricular Function in Type 2 Diabetes Mellitus: Assessment Using Two‐Dimensional Speckle Tracking Echocardiography,” Journal of Diabetes and its Complications 36, no. 5 (2022): 108167. 35272930 10.1016/j.jdiacomp.2022.108167

[2] Y. Zhang , Y. Li , Y. Lin , et al., “Association of Glycemic Control With Right Ventricular Function Assessed by Two‐Dimensional Speckle‐Tracking and Three‐Dimensional Echocardiography in Type 2 Diabetes Mellitus,” Journal of the American Society of Echocardiography 37 (2024): 1156–1166. 39278576 10.1016/j.echo.2024.09.002

[3] M. Zakir , N. Ahuja , M. A. Surksha , et al., “Cardiovascular Complications of Diabetes: From Microvascular to Macrovascular Pathways,” Cureus 15, no. 9 (2023): e45835. 37881393 10.7759/cureus.45835 PMC10594042

[4] N. Ghosh , L. Chacko , H. Bhattacharya , et al., “Exploring the Complex Relationship Between Diabetes and Cardiovascular Complications: Understanding Diabetic Cardiomyopathy and Promising Therapies,” Biomedicines 11, no. 4 (2023): 1126. 37189744 10.3390/biomedicines11041126 PMC10135805

[5] F. R. Prandi , I. Evangelista , D. Sergi , A. Palazzuoli , and F. Romeo , “Mechanisms of Cardiac Dysfunction in Diabetic Cardiomyopathy: Molecular Abnormalities and Phenotypical Variants,” Heart Failure Reviews 28, no. 3 (2023): 597–606. 35001338 10.1007/s10741-021-10200-y

[6] Y. Chen , Y. Wang , Y. Zhang , et al., “Association of Peripheral Neuropathy With Subclinical Left Ventricular Dysfunction in Patients With Type 2 Diabetes,” Journal of Diabetes and its Complications 37, no. 2 (2023): 108406. 36682230 10.1016/j.jdiacomp.2023.108406

[7] M. T. Jensen , P. Sogaard , I. Gustafsson , et al., “Echocardiography Improves Prediction of Major Adverse Cardiovascular Events in a Population With Type 1 Diabetes and Without Known Heart Disease: The Thousand & 1 Study,” Diabetologia 62 (2019): 2354–2364. 31664481 10.1007/s00125-019-05009-2

[8] S.‐M. Ghoreyshi‐Hefzabad , P. Jeyaprakash , H. Q. Vo , et al., “Subclinical Systolic Dysfunction Detected by 2D Speckle Tracking Echocardiography in Adults With Diabetes Mellitus: Systematic Review and Meta‐Analysis of 6668 Individuals With Diabetes Mellitus and 7218 Controls,” International Journal of Cardiovascular Imaging 39, no. 5 (2023): 977–989. 36995526 10.1007/s10554-023-02810-4 PMC10160195

[9] M. Berceanu , O. Mirea , I. Donoiu , C. Militaru , A. Săftoiu , and O. Istrătoaie , “Myocardial Function Assessed by Multi‐Layered Two‐Dimensional Speckle Tracking Analysis in Asymptomatic Young Subjects With Diabetes Mellitus Type 1,” Cardiology 145, no. 2 (2020): 80–87. 31825945 10.1159/000504532

[10] T. Wu , X. Li , D. Zhang , and L. G. Gong , “Early Impairment of Right Ventricular Systolic Function in Patients With Prediabetes and Type 2 Diabetes Mellitus: An Analysis of Two‐Dimensional Speckle Tracking Echocardiography,” Echocardiography 40, no. 8 (2023): 831–840. 37449864 10.1111/echo.15650

[11] X. Song , P. Zhang , L. Fan , and Y. Rui , “Epicardial Adipose Tissue and Right Ventricular Function in Type 2 Diabetes Mellitus Using Two‐Dimensional Speckle Tracking Echocardiography,” Diabetes and Vascular Disease Research 19, no. 4 (2022): 14791641221118622. 35999047 10.1177/14791641221118622 PMC9421037

[12] W. Chang , Y. Wang , L. Sun , D. Yu , Y. Li , and G. Li , “Evaluation of Left Atrial Function in Type 2 Diabetes Mellitus Patients With Nonalcoholic Fatty Liver Disease by Two‐Dimensional Speckle Tracking Echocardiography,” Echocardiography 36, no. 7 (2019): 1290–1297. 31206765 10.1111/echo.14400

[13] M. M. Łukawska‐Tatarczuk , A. Pawlak , J. Zieliński , E. Franek , L. Czupryniak , and B. Mrozikiewicz‐Rakowska , “Association of Antithyroid Peroxidase Antibodies With Cardiac Function in Euthyroid Women With Type 1 Diabetes Mellitus—Assessment With Two‐Dimensional Speckle‑Tracking Echocardiography,” Endokrynologia Polska 73, no. 5 (2022): 812–822. 35971937 10.5603/EP.a2022.0041

[14] L. Hamabe , A. S. Mandour , K. Shimada , et al., “Role of Two‐Dimensional Speckle‐Tracking Echocardiography in Early Detection of Left Ventricular Dysfunction in Dogs,” Animals: An Open Access Journal from MDPI 11, no. 8 (2021): 2361. 34438818 10.3390/ani11082361 PMC8388726

[15] L. Longobardo , V. Suma , R. Jain , et al., “Role of Two‐Dimensional Speckle‐Tracking Echocardiography Strain in the Assessment of Right Ventricular Systolic Function and Comparison With Conventional Parameters,” Journal of the American Society of Echocardiography 30, no. 10 (2017): 937–946. e6. 28803684 10.1016/j.echo.2017.06.016

[16] A. A. G. Tantawy , N. H. K. Elsherif , N. M. Habeeb , E. M. Hasan , and A. E. Abdelhameed , “A Two‐Dimensional Speckle‐Tracking Echocardiography for the Diagnosis of Early Myocardial Disease in Beta‐Thalassemia Major Patients,” Annals of Pediatric Cardiology 15, no. 3 (2022): 257–265. 36589651 10.4103/apc.apc_91_21 PMC9802624

[17] T. Haque , “Myocardial Strain Imaging Using Two and Three‐Dimensional Speckle Tracking Echocardiography: Clinical Applications,” Cardiovascular Journal 11, no. 2 (2019): 167–182.

[18] L. Martini , M. Lisi , M. C. Pastore , et al., “The Role of Speckle Tracking Echocardiography in the Evaluation of Advanced‐Heart‐Failure Patients,” Journal of Clinical Medicine 13, no. 14 (2024): 4037. 39064077 10.3390/jcm13144037 PMC11277875

[19] M. G. M. A.‐E. Alsayed , “Clinical Applications of Left Ventricular Global Longitudinal Strain by 2D‐Speckle Tracking Echocardiography,” Tobacco Regulatory Science (TRS) 9 (2023): 4825–4839.

[20] Y. Li , T. Wang , P. Haines , et al., “Prognostic Value of Right Ventricular Two‐Dimensional and Three‐Dimensional Speckle‐Tracking Strain in Pulmonary Arterial Hypertension: Superiority of Longitudinal Strain Over Circumferential and Radial Strain,” Journal of the American Society of Echocardiography 33, no. 8 (2020): 985–994. e1. 32532643 10.1016/j.echo.2020.03.015

[21] M. Hadadi , R. Mohseni‐Badalabadi , and A. Hosseinsabet , “Effects of Obesity on Left Atrial Phasic Functions in Patients With Chronic Ischemic Heart Disease and Preserved Left Ventricular Ejection Fraction Without Recent Myocardial Infarction: A Two‐Dimensional Speckle‐Tracking Echocardiography Study,” Journal of Ultrasound 25, no. 3 (2022): 521–527. 34855185 10.1007/s40477-021-00616-5 PMC9402816

[22] R. Blessing , I. Drosos , M. Molitor , et al., “Evaluation of Right‐Ventricular Function by Two‐Dimensional Echocardiography and Two‐Dimensional Speckle‐Tracking Echocardiography in Patients With Successful RCA CTO Recanalization,” Clinical Research in Cardiology 112, no. 10 (2023): 1454–1462. 37526696 10.1007/s00392-023-02259-4 PMC10562279

[23] R. Iacobelli , A. Di Molfetta , F. Cobianchi Bellisari , et al., “Changes in Left and Right Ventricular Two‐Dimensional Echocardiographic Speckle‐Tracking Indices in Pediatric LVAD Population: A Retrospective Clinical Study,” International Journal of Artificial Organs 42, no. 12 (2019): 711–716. 31238772 10.1177/0391398819857446

[24] H. Kamel , A. T. Elsayegh , H. Nazmi , and H. M. Attia , “Assessment of Left Ventricular Systolic Function Using Two‐and Three‐Dimensional Speckle Tracking Echocardiography Among Healthy Preschool‐Age Pediatric Children,” Egyptian Heart Journal 74, no. 1 (2022): 21. 10.1186/s43044-022-00258-w PMC8960099 35347471

[25] B. Yaman , O. Akpınar , H. S. Kemal , et al., “The Beneficial Effect of Low‐Intensity Exercise on Cardiac Performance Assessed by Two‐Dimensional Speckle Tracking Echocardiography,” Echocardiography 37, no. 12 (2020): 1989–1999. 33070385 10.1111/echo.14891

[26] H. Blessberger and T. Binder , “Two Dimensional Speckle Tracking Echocardiography: Basic Principles,” Heart 96, no. 9 (2010): 716–722. 20424157 10.1136/hrt.2007.141002

[27] A. Popielarz‐Grygalewicz , M. Stelmachowska‐Banaś , J. Gęsior , M. Czubalska , W. Zgliczyński , and W. Kochman , “The Influence of Acromegaly Treatment on Subclinical Left Ventricular Dysfunction Assessed by Two‐Dimensional Speckle Tracking Echocardiography (2D‐STE)‐Preliminary Results,” in Endocrine Abstracts (Bioscientifica, 2019).

[28] A. Popielarz‐Grygalewicz , M. Stelmachowska‐Banaś , J. Gęsior , M. Czubalska , W. Zgliczyński , and W. Kochman , “Subclinical Left Ventricular Dysfunction in Patients With Naive Acromegaly Assessed by Two‐Dimensional Speckle Tracking Echocardiography (2D‐STE),” in Endocrine Abstracts (Bioscientifica, 2018). 10.5603/EP.a2020.0021 32293699

[29] M. C. Arciniegas Calle , N. P. Sandhu , H. Xia , et al., “Two‐Dimensional Speckle Tracking Echocardiography Predicts Early Subclinical Cardiotoxicity Associated With Anthracycline‐Trastuzumab Chemotherapy in Patients With Breast Cancer,” BMC Cancer 18 (2018): 1037. 30359235 10.1186/s12885-018-4935-z PMC6203211

[30] W. Liu , W. Li , H. Li , et al., “Two‐Dimensional Speckle Tracking Echocardiography Help Identify Breast Cancer Therapeutics–Related Cardiac Dysfunction,” BMC Cardiovascular Disorders 22, no. 1 (2022): 548. 36522712 10.1186/s12872-022-03007-8 PMC9753263

[31] R. M. Abdulrahman , V. Delgado , A. C. T. Ng , et al., “Abnormal Cardiac Contractility in Long‐Term Exogenous Subclinical Hyperthyroid Patients as Demonstrated by Two‐Dimensional Echocardiography Speckle Tracking Imaging,” European Journal of Endocrinology 163, no. 3 (2010): 435–441. 20587582 10.1530/EJE-10-0328

[32] X. Tao , M. Jiang , Y. Liu , et al., “Predicting Three‐Month Fasting Blood Glucose and Glycated Hemoglobin Changes in Patients With Type 2 Diabetes Mellitus Based on Multiple Machine Learning Algorithms,” Scientific Reports 13, no. 1 (2023): 16437. 37777593 10.1038/s41598-023-43240-5 PMC10543442

[33] M. Tadic , N. Nita , L. Schneider , et al., “The Predictive Value of Right Ventricular Longitudinal Strain in Pulmonary Hypertension, Heart Failure, and Valvular Diseases,” Frontiers in Cardiovascular Medicine 8 (2021): 698158. 34222387 10.3389/fcvm.2021.698158 PMC8247437

[34] H. Motoki , A. G. Borowski , K. Shrestha , et al., “Right Ventricular Global Longitudinal Strain Provides Prognostic Value Incremental to Left Ventricular Ejection Fraction in Patients With Heart Failure,” Journal of the American Society of Echocardiography 27, no. 7 (2014): 726–732. 24679740 10.1016/j.echo.2014.02.007

[35] R. Wilson , S. Eguchi , Y. Orihara , et al., “Association Between Right Ventricular Global Longitudinal Strain and Mortality in Intermediate‐Risk Pulmonary Embolism,” Echocardiography 41, no. 4 (2024): e15815. 38634182 10.1111/echo.15815

[36] H. Usuku , E. Yamamoto , D. Sueta , et al., “Prognostic Value of Right Ventricular Global Longitudinal Strain in Patients With Immunoglobulin Light‐Chain Cardiac Amyloidosis,” European Heart Journal Open 3, no. 3 (2023): oead048. 37214543 10.1093/ehjopen/oead048 PMC10196102

[37] S. L. Purwowiyoto and R. Halomoan , “Highlighting the Role of Global Longitudinal Strain Assessment in Valvular Heart Disease,” Egyptian Heart Journal 74, no. 1 (2022): 46. 10.1186/s43044-022-00283-9 PMC9156579 35639183

[38] C. Li , X. Liu , B. K. Adhikari , et al., “The Role of Epicardial Adipose Tissue Dysfunction in Cardiovascular Diseases: An Overview of Pathophysiology, Evaluation, and Management,” Frontiers in Endocrinology 14 (2023): 1167952. 37260440 10.3389/fendo.2023.1167952 PMC10229094

[39] G. Iacobellis , “Epicardial Adipose Tissue in Contemporary Cardiology,” Nature Reviews Cardiology 19, no. 9 (2022): 593–606. 35296869 10.1038/s41569-022-00679-9 PMC8926097

[40] A. H. Talman , P. J. Psaltis , J. D. Cameron , I. T. Meredith , S. K. Seneviratne , and D. T. Wong , “Epicardial Adipose Tissue: Far More Than a Fat Depot,” Cardiovascular Diagnosis and Therapy 4, no. 6 (2014): 416–429. 25610800 10.3978/j.issn.2223-3652.2014.11.05 PMC4278038

[41] A. M. Ansaldo , F. Montecucco , A. Sahebkar , F. Dallegri , and F. Carbone , “Epicardial Adipose Tissue and Cardiovascular Diseases,” International Journal of Cardiology 278 (2019): 254–260. 30297191 10.1016/j.ijcard.2018.09.089

[42] Y. Song , Y. Tan , M. Deng , et al., “Epicardial Adipose Tissue, Metabolic Disorders, and Cardiovascular Diseases: Recent Advances Classified by Research Methodologies,” MedComm 4, no. 6 (2023): e413. 37881786 10.1002/mco2.413 PMC10594046

[43] J. Xu , Y. Xue , Q. Chen , et al., “Identifying Distinct Risk Thresholds of Glycated Hemoglobin and Systolic Blood Pressure for Rapid Albuminuria Progression in Type 2 Diabetes From NHANES (1999–2018),” Frontiers in Medicine 9 (2022): 928825. 35795642 10.3389/fmed.2022.928825 PMC9251013

[44] X. Huang , C. Qin , X. Guo , F. Cao , and C. Tang , “Association of Hemoglobin A1c With the Incidence of Hypertension: A Large Prospective Study,” Frontiers in Endocrinology 13 (2023): 1098012. 36726461 10.3389/fendo.2022.1098012 PMC9884972

[45] T. Takao , Y. Matsuyama , M. Suka , H. Yanagisawa , and Y. Iwamoto , “The Combined Effect of Visit‐to‐Visit Variability in HbA1c and Systolic Blood Pressure on the Incidence of Cardiovascular Events in Patients With Type 2 Diabetes,” BMJ Open Diabetes Research & Care 3, no. 1 (2015): e000129. 10.1136/bmjdrc-2015-000129 PMC4653863 26629346

[46] M. Kosmalski , S. Ziółkowska , P. Czarny , J. Szemraj , and T. Pietras , “The Coexistence of Nonalcoholic Fatty Liver Disease and Type 2 Diabetes Mellitus,” Journal of Clinical Medicine 11, no. 5 (2022): 1375. 35268466 10.3390/jcm11051375 PMC8910939

[47] C.‐T. Diaconu and C. Guja , “Nonalcoholic Fatty Liver Disease and Its Complex Relation With Type 2 Diabetes Mellitus—From Prevalence to Diagnostic Approach and Treatment Strategies,” Journal of Clinical Medicine 11, no. 17 (2022): 5144. 36079070 10.3390/jcm11175144 PMC9456683

[48] J. Huang , H. L. Hu , Z. N. Yan , et al., “Peak Systolic Longitudinal Rotation: A New Tool for Detecting Left Ventricular Systolic Function in Patients With Type 2 Diabetes Mellitus by Two‐Dimensional Speckle Tracking Echocardiography,” BMC Cardiovascular Disorders 19 (2019): 137. 31174469 10.1186/s12872-019-1119-y PMC6556012

[49] J. Zhu , W. Li , F. Chen , Z. Xie , K. Zhuo , and R. Huang , “Impact of Glycemic Control on Biventricular Function in Patients With Type 2 Diabetes Mellitus: A Cardiac Magnetic Resonance Tissue Tracking Study,” Insights Into Imaging 14, no. 1 (2023): 7. 36630007 10.1186/s13244-022-01357-7 PMC9833026

[50] D. Carvalho , R. Haaverstad , B. Cumberledge , et al., “Dobutamine Stress Echocardiography for Assessment of Myocardial Viability: Impact of Beta‐Blockade on Exam Accuracy,” Cardiology Letters 87, no. 4 (2024): 329–335.

[51] H. Ismail , J. K. Gabriels , D. Chang , et al., “Site‐Specific Effects of Dobutamine on Cardiac Conduction and Refractoriness,” Journal of Interventional Cardiac Electrophysiology 67, no. 1 (2024): 71–82. 37227538 10.1007/s10840-023-01573-1

[52] M. G. Mousa , I. M. R. Faheem , A. F. Elkhateeb , M. A. Saad , and K. A. E. El‐Khashab , “Dobutamine Stress Echocardiography in Patients With Chronic Peripheral Artery Disease,” International Journal of Health Sciences 6, no. S7 (2023): 6974–6990.

[53] C. Philouze , P. Obert , S. Nottin , A. Benamor , O. Barthez , and F. Aboukhoudir , “Dobutamine Stress Echocardiography Unmasks Early Left Ventricular Dysfunction in Asymptomatic Patients With Uncomplicated Type 2 Diabetes: A Comprehensive Two‐Dimensional Speckle‐Tracking Imaging Study,” Journal of the American Society of Echocardiography 31, no. 5 (2018): 587–597. 29526563 10.1016/j.echo.2017.12.006

[54] Y. Tian , Y. Zhang , Q. Yan , et al., “Fragmented QRS Complex in Healthy Adults: Prevalence, Characteristics, Mechanisms, and Clinical Implications,” International Journal of Heart Rhythm 2, no. 1 (2017): 34–39.

[55] A. C. Ratheendran , M. Subramanian , D. K. Bhanu , et al., “Fragmented QRS on Electrocardiography as a Predictor of Myocardial Scar in Patients With Hypertrophic Cardiomyopathy,” Acta Cardiologica 75 (2020): 42–46. 30602338 10.1080/00015385.2018.1547355

[56] M. K. Das , B. Khan , S. Jacob , A. Kumar , and J. Mahenthiran , “Significance of a Fragmented QRS Complex Versus a Q Wave in Patients With Coronary Artery Disease,” Circulation 113, no. 21 (2006): 2495–2501. 16717150 10.1161/CIRCULATIONAHA.105.595892

[57] N. Engstrom , G. Dobson , K. Ng , and H. Letson , “Fragmented QRS Is Associated With Ventricular Arrhythmias in Heart Failure Patients: A Systematic Review and Meta‐Analysis,” Annals of Noninvasive Electrocardiology 27, no. 1 (2022): e12910. 34766402 10.1111/anec.12910 PMC8739614

[58] A. Bayramoğlu , H. Taşolar , Y. Kaya , et al., “Fragmented QRS Complexes Are Associated With Left Ventricular Dysfunction in Patients With Type‐2 Diabetes Mellitus: A Two‐Dimensional Speckle Tracking Echocardiography Study,” Acta Cardiologica 73, no. 5 (2018): 449–456. 29216794 10.1080/00015385.2017.1410350

[59] A. Brand , E. Romero Dorta , A. Wolf , et al., “Phasic Left Atrial Strain to Predict Worsening of Diastolic Function: Results From the Prospective Berlin Female Risk Evaluation Follow‐Up Trial,” Frontiers in Cardiovascular Medicine 10 (2023): 1070450. 36891246 10.3389/fcvm.2023.1070450 PMC9986257

[60] J. Cai , Z. Liang , W. Feng , and H. Long , “Correlation Between Left Atrial Strain and Left Ventricular Diastolic Function in Hypertensive Patients,” Zhong Nan Da Xue Xue Bao. Yi Xue Ban = Journal of Central South University 48, no. 6 (2023): 846–851. 10.11817/j.issn.1672-7347.2023.220301 PMC10930438 37587069

[61] E. Romero Dorta , A. Wolf , A. Hübscher , et al., “Impact of Body Mass Index on Worsening of Diastolic Function and Impairment of Left Atrial Strain in the General Female Urban Population: A Subanalysis of the Berlin Female Risk Evaluation Echocardiography Follow‐Up Study,” Frontiers in Cardiovascular Medicine 10 (2023): 1242805. 37799777 10.3389/fcvm.2023.1242805 PMC10548209

[62] J. Liu , J. Li , C. Xia , et al., “Diastolic Dysfunction in Adults With Uncomplicated Obesity Evaluated With Left Atrial and Left Ventricular Tissue Tracking and Ventricular Volume‐Time Curve: A Prospective Cardiac Magnetic Resonance Study,” Quantitative Imaging in Medicine and Surgery 14, no. 7 (2024): 5040–5056. 39022235 10.21037/qims-23-1785 PMC11250294

[63] M. Barki , M. Losito , M. M. Caracciolo , et al., “Left Atrial Strain in Acute Heart Failure: Clinical and Prognostic Insights,” European Heart Journal‐Cardiovascular Imaging 25, no. 3 (2024): 315–324. 37930715 10.1093/ehjci/jead287

[64] A. Hosseinsabet , R. Mohseni‐Badalabadi , and A. Moinfar , “Impaired Left Atrial Conduit Function in Coronary Artery Disease Patients With Poorly Controlled Diabetes: Two‐Dimensional Speckle‐Tracking Echocardiographic Study,” Journal of Ultrasound in Medicine 36, no. 1 (2017): 13–23. 27925659 10.7863/ultra.15.12065

[65] L.‐C. Benchea , L. Anghel , A. Zăvoi , et al., “Beyond Blood Sugar: How Left Atrium Strain Predicts Cardiac Outcomes in Type 2 Diabetes,” Biomedicines 12, no. 8 (2024): 1690. 39200155 10.3390/biomedicines12081690 PMC11351471

[66] G. Mandoli , M. Pastore , G. Benfari , et al., “Pathophysiologic Risk Stratification of Chronic Heart Failure: Coexisting Left Atrial and Right Ventricular Damage and the Role of Pulmonary Circulation,” supplement, European Heart Journal‐Cardiovascular Imaging 22, no. S1 (2021): jeaa356. 180.

[67] S. Wang , C. Cui , Y. Li , et al., “Interaction Effect of Type 2 Diabetes Mellitus and Hypertension on Left Atrial Function: A Three‐Dimensional Echocardiography Study,” Quantitative Imaging in Medicine and Surgery 13, no. 12 (2023): 8107–8120. 38106252 10.21037/qims-23-795 PMC10722055

[68] T. G. Utina , D. U. Akasheva , D. V. Korsunsky , and O. M. Drapkina , “Significance of Standard and Speckle‐Tracking Echocardiography for Early Diagnosis of Asymptomatic Left Ventricular Dysfunction in Type 2 Diabetes,” Cardiovascular Therapy and Prevention 22, no. 1 (2023): 3478.

[69] L. Georgievska‐Ismail , P. Zafirovska , and Z. Hristovski , “Evaluation of the Role of Left Atrial Strain Using Two‐Dimensional Speckle Tracking Echocardiography in Patients With Diabetes Mellitus and Heart Failure With Preserved Left Ventricular Ejection Fraction,” Diabetes and Vascular Disease Research 13, no. 6 (2016): 384–394. 27407084 10.1177/1479164116655558

[70] A. Goyal , H. Q. Abbasi , S. Yakkali , et al., “Left Atrial Strain as a Predictor of Early Anthracycline‐Induced Chemotherapy‐Related Cardiac Dysfunction: A Pilot Systematic Review and Meta‐Analysis,” Journal of Clinical Medicine 13, no. 13 (2024): 3904. 38999470 10.3390/jcm13133904 PMC11242155

[71] Y. Su , C. Li , and L. Yin , “Assessment the Predictive Value of Left Atrial Strain (LAS) on Exercise Tolerance in HCM Patients With E/e' Between 8 and 14 by Two‐Dimensional Speckle Tracking and Treadmill Stress Echocardiography,” Reviews in Cardiovascular Medicine 24, no. 6 (2023): 167. 39077539 10.31083/j.rcm2406167 PMC11264115

[72] E. Pilichowska‐Paszkiet , A. Sikorska , I. Kowalik , et al., “Subclinical Dysfunction of Left Atrial Compliance After Cryoballoon Versus Radiofrequency Ablation for Paroxysmal Atrial Fibrillation,” Journal of Clinical Medicine 12, no. 15 (2023): 4974. 37568376 10.3390/jcm12154974 PMC10420106

[73] Y. Liu , K. Wang , D. Su , et al., “Noninvasive Assessment of Left Atrial Phasic Function in Patients With Hypertension and Diabetes Using Two‐Dimensional Speckle Tracking and Volumetric Parameters,” Echocardiography 31, no. 6 (2014): 727–735. 24354465 10.1111/echo.12492

[74] T. A. Ahmed , Y. A. Ahmed , A. I. Arafa , and R. A. Salah , “Detection of Occult Right Ventricular Dysfunction in Young Egyptians With Type 1 Diabetes Mellitus by Two‐Dimensional Speckle Tracking Echocardiography,” Indian Heart Journal 70, no. 5 (2018): 665–671. 30392504 10.1016/j.ihj.2018.06.019 PMC6204469

[75] S. Rakha and H. M. Aboelenin , “Left Ventricular Functions in Pediatric Patients With Ten Years or More Type 1 Diabetes Mellitus: Conventional Echocardiography, Tissue Doppler, and Two‐Dimensional Speckle Tracking Study,” Pediatric Diabetes 20, no. 7 (2019): 946–954. 31355962 10.1111/pedi.12900

[76] H. Wang , N. Li , T. Chivese , et al., “IDF Diabetes Atlas: Estimation of Global and Regional Gestational Diabetes Mellitus Prevalence for 2021 by International Association of Diabetes in Pregnancy Study Group's Criteria,” Diabetes Research and Clinical Practice 183 (2022): 109050. 34883186 10.1016/j.diabres.2021.109050

[77] T. Mazumder , E. Akter , S. M. Rahman , M. T. Islam , and M. R. Talukder , “Prevalence and Risk Factors of Gestational Diabetes Mellitus in Bangladesh: Findings From Demographic Health Survey 2017–2018,” International Journal of Environmental Research and Public Health 19, no. 5 (2022): 2583. 35270274 10.3390/ijerph19052583 PMC8909680

[78] W. Li , Z. Li , W. Liu , et al., “Two‐Dimensional Speckle Tracking Echocardiography in Assessing the Subclinical Myocardial Dysfunction in Patients With Gestational Diabetes Mellitus,” Cardiovascular Ultrasound 20, no. 1 (2022): 21. 35941651 10.1186/s12947-022-00292-3 PMC9361647

[79] A. Sonaglioni , E. Barlocci , G. Adda , et al., “The Impact of Short‐Term Hyperglycemia and Obesity on Biventricular and Biatrial Myocardial Function Assessed by Speckle Tracking Echocardiography in a Population of Women With Gestational Diabetes Mellitus,” Nutrition, Metabolism, and Cardiovascular Diseases 32, no. 2 (2022): 456–468. 10.1016/j.numecd.2021.10.011 34893411

